# Biocatalytic kinetic resolution of d,l-pantolactone by using a novel recombinant d-lactonase

**DOI:** 10.1039/d0ra09053k

**Published:** 2020-12-24

**Authors:** Qiu-Hua Zhang, Yi Fang, Wen-Fang Luo, Liu-Nv Huang

**Affiliations:** Brother Research Center, Jiangxi Brother Pharmaceutical Co.,Ltd Jiujiang 332700 China zqh@brother.com.cn

## Abstract

d-Pantolactone is a key chiral intermediate for the synthesis of d-pantothenic acid and its derivatives. Biocatalytic kinetic resolution of d,l-pantoyl lactone using d-lactonase is an efficient route to synthesize d-pantolactone. In this study, we report the expression of a novel d-lactonase TSDL in *Escherichia coli* host. The recombinant TSDL exhibited high hydrolysis activity and enantioselectivity toward d-pantolactone. The reaction conditions of the recombinant TSDL-catalyzed kinetic resolution of d,l-pantolactone was systematically investigated by whole cell biocatalysis. In addition, a preparative-scale reaction for bioproduction of d-pantoic acid was examined under optimized reaction conditions. This study presented an alternative enzymatic process for kinetic resolution of d,l-pantolactone.

## Abstract 2

d-Pantolactone is a key chiral intermediate for the synthesis of d-pantothenic acid and its derivatives.

## Graphical abstract

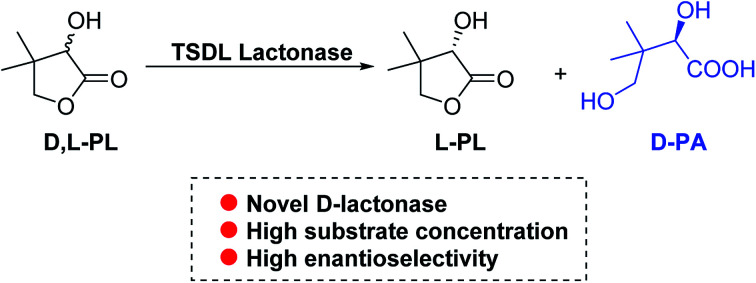

## Introduction

d-Pantolactone (d-PL) is an important chiral building block for the synthesis of d-pantothenic acid (vitamin B5) and its derivatives, which are used as ingredients in pharmaceuticals and as useful additives for animal feeds.^1^ Annual global production of calcium d-pantothenate is approximately 30 000 t. Kinetic resolution of d,l-PL is a feasible approach for the preparation of optically active d-PL. The chemical kinetic resolution processes suffer from using expensive alkaloid or chiral amine as a resolving reagent. On the contrary, enzymatic kinetic resolution of d,l-PL exhibits obvious advantages in economic and environmental aspects. Although several other enzymatic routes have been reported for the synthesis of d-PL,^2–6^ the lactonase-catalyzed kinetic resolution of d,l-PL is more practical for commercial production of d-PL.

**Fig. 1:**
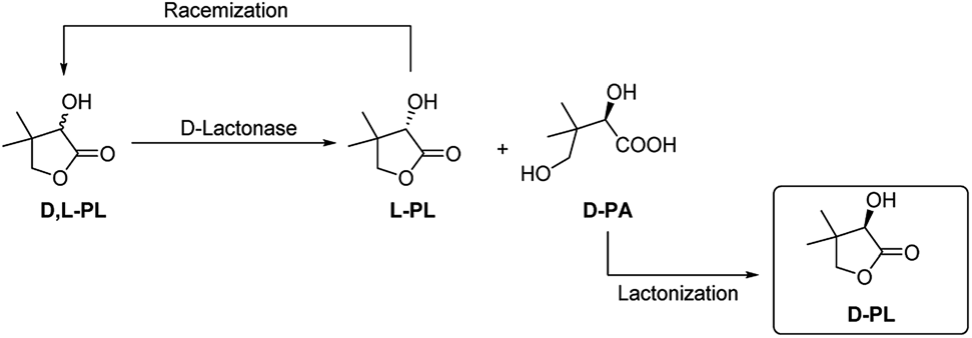
Chemoenzymatic process for the production of enantiopure d-PL.

Both of enantioselective d-lactonase and l-lactonase have been investigated for kinetic resolution of d,l-PL. Shimizu and his coworkers reported a d-lactonase (FODL) produced fungi *Fusarium oxysporum* for efficient hydrolysis of d-PL, and this biotechnology has been used for commerical production of enantiopure d-PL by Fuji/Daiichi Chemicals, Japan.^7–9^ The encode gene of FODL has been cloned and expressed in *Escherichia coli* and *Aspergillus oryzae*.^10,11^ In addition, Tang and his coworkers also developed an enzymatic resolution method by using a d-lactonase (FMDL) produced organism *Fusarium moniliforme* SW-902,^12^ which has been used for commerical preparation of d-PL by Zhejiang Xinfu Biochemical Co., Ltd, China. In 2002 Kesseler reported the heterologous expression, immobilization and directed evolution of a enantioselective l-lactonase (ATLL) from *Agrobacterium tumefaciens* Lu681.^13^ Later, Xu group reported another l-lactonase (FPLL) from *Fusarium proliferatum* ECU2002,^14^ and they further cloned the encode gene of FPLL for expression in *E. coli*.^15^ Although kinetic resolution of d,l-PL using l-specific lactonase lead to direct generation of d-PL, the optical purity of d-PL highly depends on the hydrolysis degree of l-PL. To date the chemoenzymatic process using d-lactonase is more advantageous for practical purposes (Fig. 1). In this case, the enantiopure d-pantotic acid (d-PA) generated by d-lactonase is isolated and converted to d-PL by a chemical lactonization step.

Although several other fungal strains have been found to preferentially hydrolyze the d-PL, most of these d-lactonases showed low enantioselectivity.^16^ Therefore, the finding of novel d-lactonase with high enantioselectivity for commerical use still attracts a lot of attention. In this study, a novel d-lactonase TSDL from *Thielavia* sp. zmu20201 was cloned and expressed in *E. coli*. The recombinant TSDL exhibited high hydrolysis activity and enantioselectivity toward d-PL, which was a promising biocatalyst for bioproduction of chiral d-PA.

## Results and discussion

### Expression and sequence analysis of TSDL in *E. coli*

**Fig. 2:**
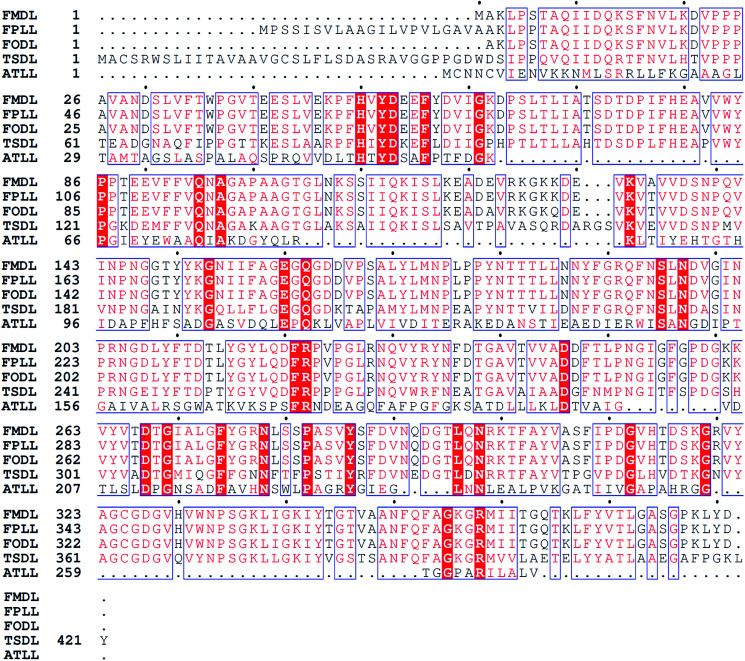
Alignment of the peptide sequences of TSDL (protein sequence ID: XP_003651251.1), FODL (protein sequence ID: BAA34062.1), FMDL (protein sequence ID: AAU50673.1), FPDL (protein sequence ID: ACC61057.1) and ATLL (protein sequence ID: WP_137411088.1).

The TSDL gene was cloned and expressed in *E. coli* BL21(DE3) using pET-28a(+) vector. The recombinant *E. coli* (TSDL) strain was screened and cultured in LB medium. The recombinant TSDL was expressed as intracellular protein by inducing with IPTG. Successful expression of TSDL was verified by SDS-PAGE analysis, which displayed a heterologous protein band about 45 kDa in *E. coli* (TSDL) strain (data not shown). The TSDL peptide was composed of 421 amino acids. Blast search in NCBI database revealed the amino acid sequence of TSDL showed 100% sequence identity to a uncharacterized protein THITE_2046186 (protein sequence ID: XP_003651251.1) from strain *Thermothielavioides terrestris* NRRL 8126. Further sequence alignment with several other lactonases was performed using Clustal W and ESPript 3.0,^17,18^ and the result was showed in Fig. 2. The TSDL shared about 61% similarity to FODL and FMDL, 58.6% similarity to FPLL and 12.6% similarity to ATLL.

### Investigation of reaction temperature for bioproduction of d-PA

**Fig. 3:**
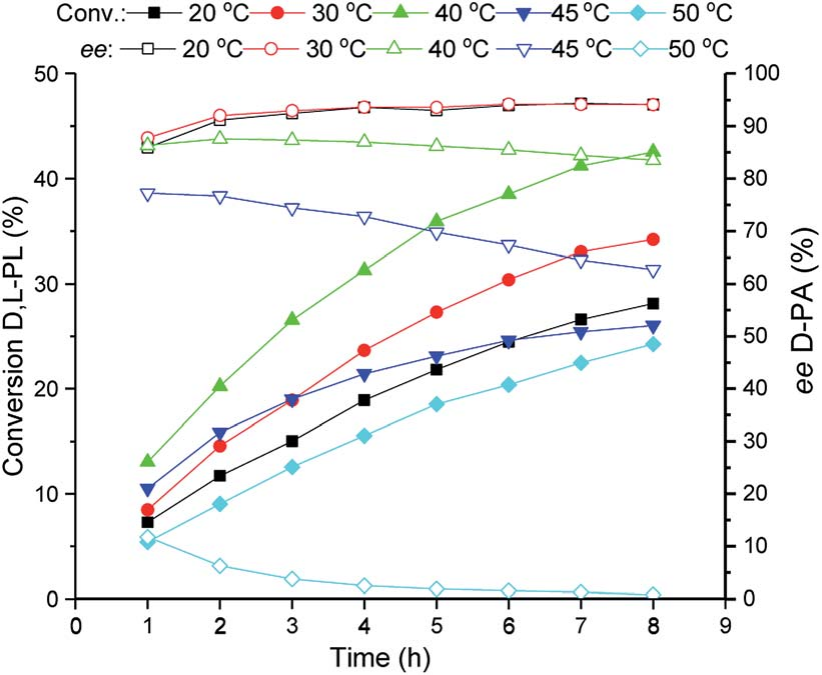
Reaction courses of kinetic resolution of d,l-PL at different temperatures. Filled symbols: conversion of d,l-PL; open symbols: *ee* of d-PA.

The reaction temperature of the TSDL-catalyzed kinetic resolution of d,l-PL was investigated, and the result was showed in Fig. 3. The hydrolysis rate was improved with the increase of reaction temperature. The maximum hydrolysis rate was found at 40 °C. Further increase of temperature to 45 or 50 °C caused dramatical decrease of hydrolysis rate, which gave about 20% conversion after 8 h. Importantly, the hydrolysis enantioselectivity decreased with the temperature increase. Reaction at 30 °C exhibited relative high conversion and good enantioselectivity, which gave 34% conversion of d,l-PL and 94% *ee* of d-PA.

### Investigation of reaction pH for bioproduction of d-PA

**Fig. 4:**
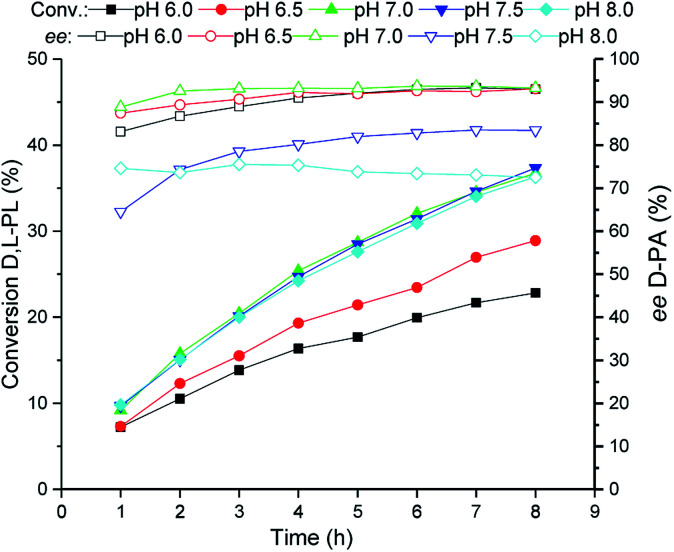
Reaction courses of kinetic resolution of d,l-PL at different pH. Filled symbols: conversion of d,l-PL; open symbols: *ee* of d-PA.

The reaction pH of the TSDL-catalyzed kinetic resolution of d,l-PL was then examined. As shown in Fig. 4, the hydrolysis rate was improved when the reaction pH increased from 6.0 to 7.0. Further increase of pH did not lead to enhancement of hydrolysis activity as well as resulted in obvious decrease in enantioselectivity. Therefore, the optimum reaction pH for kinetic resolution of d,l-PL was controlled at 7.0 ± 0.2.

### Investigation of cell loading for bioproduction of d-PA

**Fig. 5:**
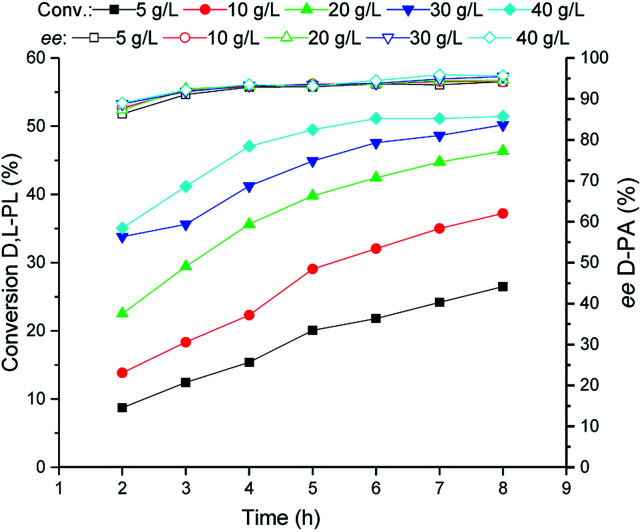
Reaction courses of kinetic resolution of d,l-PL at different cell density. Filled symbols: conversion of d,l-PL; open symbols: *ee* of d-PA.

Subsequently, the TSDL-catalyzed kinetic resolution of d,l-PL at different cell density was tested. As shown in Fig. 5, increase of cell loading leaded to improvement of hydrolysis rate. When the cell density increased to 40 g WCW per L, the hydrolysis process was finished after 5 h, which gave 50% conversion of d,l-PL and 95% *ee* of d-PA. Thus we used 40 g WCW per L *E. coli* (TSDL) cells in the following study.

### Investigation of substrate concentration for bioproduction of d-PA

**Fig. 6:**
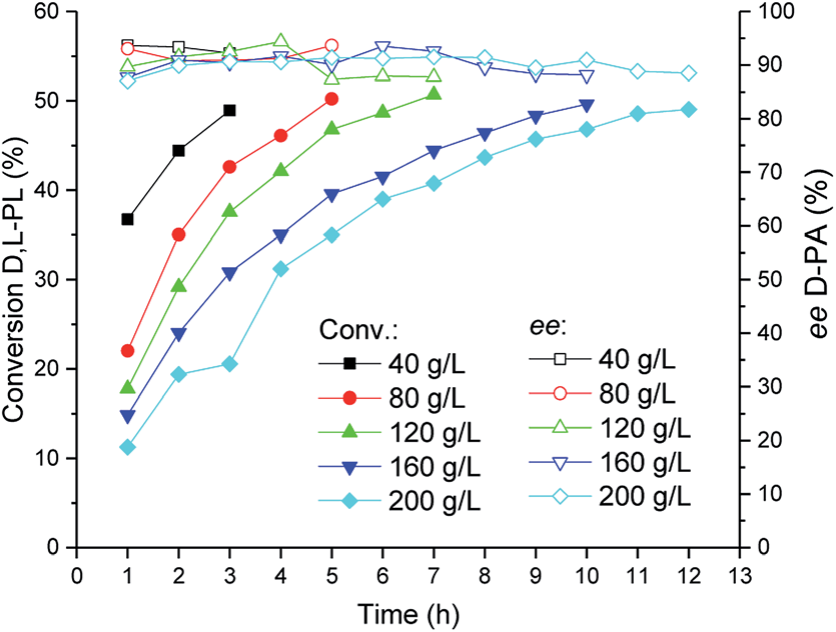
Reaction courses of kinetic resolution of d,l-PL at different substrate concentrations. Filled symbols: conversion of d,l-PL; open symbols: *ee* of d-PA.

The TSDL-catalyzed kinetic resolution of d,l-PL at different substrate concentration was investigated, and the result was showed in Fig. 6. When the substrate concentration decreased to 40 g L^−1^, the resolution process was completed in 3 h. In addition, the hydrolysis reaction could be finished after 7 and 10 h, when we increased the substrate concentration to 120 and 160 g L^−1^, respectively. To our delight, for a higher substrate concentration of 200 g L^−1^, the resolution reaction was almost completed after 12 h, which gave 49% conversion of d,l-PL and 90% *ee* of d-PA.

### Preparative-scale reaction for bioproduction of d-PA

**Fig. 7:**
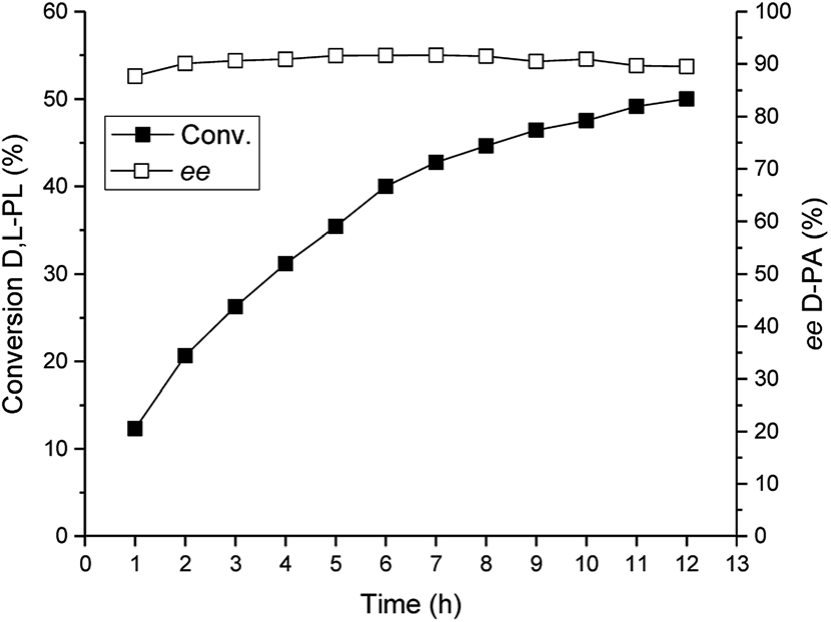
Reaction course of the TSDL-catalyzed kinetic resolution of d,l-PL in a preparative-scale system. Filled symbols: conversion of d,l-PL; open symbols: ee of d-PA.

Under the optimized reaction conditions, we performed a preparative-scale reaction for production of d-PA at a 200 g L^−1^ substrate concentration. The reaction was carried out in a 400 mL system using 16 g wet cell of *E. coli* (TSDL). The reaction course was monitored by HPLC, and the result was displayed in Fig. 7. The hydrolysis reaction was smoothly finished after 12 h, which gave 50% conversion of d,l-PL and 90% *ee* of d-PA. This result indicated that kinetic resolution of d,l-PL using TSDL in a large scale reaction system was also practicable.

## Conclusions

d-lactonase is useful in the bioproduction of d-PA *via* kinetic resolution of d,l-PL. However, the known d-lactonase with high enantioselectivity is very few until now. In addition, the d-lactonase usually comes from fungal strains such as *Fusarium*, and involves in the complicated post-translational modifications, for instance, *N*-glycosylation. These post-translational modifications were absent in prokaryotic organisms, which resulted in that efficient expression of d-lactonase in prokaryotic host cell was difficult. For example, Honda has demonstrated that expression of the lactonase FODL in *E. coli* host was not sufficient.^11^

In this study, we reported a novel enantioselective d-lactonase of TSDL from *Thielavia* sp. zmu20201, which exhibited high enantioselectivity toward d-PL. Interestingly, the TSDL was suitable for expression in *E. coli* host. The recombinant *E. coli* (TSDL) strain was constructed, and we systematically investigated the kinetic resolution process of d,l-PL using the whole cell as the biocatalyst. We found that high temperature and pH highly influenced the hydrolysis activity as well as enantioselectivity. The optimum temperature and pH for the hydrolysis of d-PL were 30 °C and pH 7.0, respectively. By futher optimization of cell loading and substrate concentration, the biocatalytic kinetic resolution of d,l-PL process using whole cell of TSDL was developed, which was carried out in 200 g L^−1^ of d,l-PL with 40 g L^−1^ WCW per L of recombinant *E. coli* (TSDL) cells. To verify the synthetic ability of this biocatalytic process, a preparative-scale reaction was performed to obtain high conversion and good enantioselectivity. These results indicated the TSDL was a promising biocatalyst for commercial production of chiral d-PL.

In summary, we have cloned and expressed a novel d-lactonase TSDL in *E. coli* host. The recombinant *E. coli* (TSDL) strain exhibited high hydrolysis activity and enantioselectivity in kinetic resolution of d,l-PL, providing a practical synthetic method for biocatalytic production of d-PA.

## Experimental

### Cloning and expression of TSDL

The pGEM-TSDL plasmid inserted with lactonase TSDL gene was donated by Dr Wan from Zunyi Medical University, which was cloned from organism *Thielavia* sp. zmu20201(CGMCC No. 2020062). The TSDL encode gene was cloned into pET-28a(+) plasmid by *Nco* I and *Xho* I enzyme sites. The recombinant plasmid pET-28-TSDL was then transformed into *E. coli* BL(DE3) and the positive *E. coli* (TSDL) strain harboring the plasmid pET-28-TSDL was screened by colony PCR.

For expression of recombinant TSDL, single colony of *E. coli* (TSDL) strain was picked and inoculated to 10 mL LB medium (tryptone, 10 g L^−1^; yeast extract, 5 g L^−1^; NaCl, 10 g L^−1^) containing 50 μg mL^−1^ kanamycin. After incubation at 37 °C for 12 h, 5 mL culture was taken and transferred into 100 mL fresh LB medium. The culture was incubated at 37 °C until the OD_600_ up to 0.6–0.8. To the culture isopropyl-β-*d*-thiogalactoside (IPTG) was added to the final concentration of 0.2 mM, and the culture was incubated at 28 °C for expression of TSDL for another 12–14 h. Then the *E. coli* (TSDL) cells were harvested by centrifugation at 9000×*g*, 4 °C for 10 min. The harvested cells was stored at −20 °C for further use.

### Investigation of reaction temperature for bioproduction of d-PA

Reaction temperature of the biocatalytic kinetic resolution of d,l-PL was investigated at 20, 30, 40, 45 and 50 °C. Reactions were carried out in 20 mL water containing 80 g L^−1^d,l-PL and 10 g WCW per L recombinant *E. coli* (TSDL) cells. The pH was controlled at 7.0 ± 0.2 using 5% NH_3_·H_2_O. The reaction courses were monitored by taking samples from the mixtures. After removal of the cells by centrifugation, the conversion of d,l-PL and the optical purity of product d-PA were determined by HPLC analysis.

### Investigation of reaction pH for bioproduction of d-PA

Reaction pH of the biocatalytic kinetic resolution of d,l-PL was investigated at 30 °C. The pH values in each independent reaction were controlled at 6.0 ± 0.2, 6.5 ± 0.2, 7.0 ± 0.2, 7.5 ± 0.2, 8.0 ± 0.2 and 8.5 ± 0.2 using 5% NH_3_·H_2_O. Reactions were carried out in 20 mL water containing 80 g L^−1^d,l-PL and 10 g WCW per L recombinant *E. coli* (TSDL) cells. The reaction courses were monitored by taking samples from the mixtures. After removal of the cells by centrifugation, the conversion of d,l-PL and the optical purity of product d-PA were determined by HPLC analysis.

### Investigation of cell loading for bioproduction of d-PA

Biocatalyst loading of the biocatalytic kinetic resolution of d,l-PL was investigated at 30 °C. Reactions were carried out in 20 mL water containing 80 g L^−1^d,l-PL and the reaction pH was controlled at 7.0 ± 0.2 using 5% NH_3_·H_2_O. Cell concentrations of recombinant *E. coli* (TSDL) cells were set at 5, 10, 20, 30 and 40 g WCW per L. The reaction courses were monitored by taking samples from the mixtures. After removal of the cells by centrifugation, the conversion of d,l-PL and the optical purity of product d-PA were determined by HPLC analysis.

### Investigation of substrate concentration for bioproduction of d-PA

Substrate concentration of the biocatalytic kinetic resolution of d,l-PL was investigated at 30 °C and pH 7.0 ± 0.2. Reactions were carried out in 20 mL water containing 40 g WCW per L recombinant *E. coli* (TSDL) cells and different substrate concentrations (40, 80, 120, 160 and 200 g L^−1^) of d,l-PL. The reaction courses were monitored by taking samples from the mixtures. After removal of the cells by centrifugation, the conversion of d,l-PL and the optical purity of product d-PA were determined by HPLC analysis.

### Preparative-scale for bioproduction of d-PA

The preparative-scale reaction of the biocatalytic kinetic resolution of d,l-PL was carried out in 400 mL water containing 200 g L^−1^d,l-PL and 40 g WCW per L recombinant *E. coli* (TSDL) cells. The reaction was performed at 30 °C and the reaction pH was controlled at 7.0 ± 0.2 using 5% NH_3_·H_2_O. The reaction course was monitored by taking samples from the reaction mixture. After removal of the cells by centrifugation, the conversion of d,l-PL and the optical purity of product d-PA were determined by HPLC analysis.

### Analytic methods

The conversion of d,l-PL was estimated by HPLC on an ODS-3 column (4.6 × 250 mm, 5 μm, Shimadzu, Tokyo, Japan) at 210 nm with a mobile phase of 10% acetonitrile containing 0.02 mM KH_2_PO_4_ with a flow rate of 1.0 mL min^−1^. The retention times of d,l-PL and d,l-PA were 4.92 and 8.89 min respectively. The optical purity of d-PA was determined by HPLC on an MCI GEL CRS10W packed column (4.6 × 50 mm, 3 μm, Mitsubishi Kasei, Tokyo, Japan) at 254 nm with a mobile phase of 10% acetonitrile containing 1.8 mM CuSO_4_ with a flow rate of 0.8 mL min^−1^. The retention times of d-PA and l-PA were 2.82 and 3.75 min respectively.

## Conflicts of interest

There are no conflicts to declare.

## Supplementary Material

## References

[cit1] Shimizu S. , Biotechnology: Special Processes, Wiley-VCH, 2001, pp. 318–340

[cit2] Si D., Urano N., Shimizu S., Kataoka M. (2012). Appl. Microbiol. Biotechnol..

[cit3] Zhao M., Gao L., Zhang L., Bai Y., Chen L., Yu M., Cheng F., Sun J., Wang Z., Ying X. (2017). Biotechnol. Lett..

[cit4] Heidlindemann M., Hammel M., Scheffler U., Mahrwald R., Hummel W., Berkessel A., Gröger H. (2015). J. Org. Chem..

[cit5] Si D., Urano N., Nozaki S., Honda K., Shimizu S., Kataoka M. (2012). Appl. Microbiol. Biotechnol..

[cit6] Pscheidt B., Avi M., Gaisberger R., Hartner F. S., Skranc W., Glieder A. (2008). J. Mol. Catal. B Enzym..

[cit7] Kataoka M., Shimizu K., Sakamoto K., Yamada H., Shimizu S. (1995). Appl. Microbiol. Biotechnol..

[cit8] Kataoka M., Shimizu K., Sakamoto K., Yamada H., Shimizu S. (1995). Appl. Microbiol. Biotechnol..

[cit9] Sakamoto K., Honda K., Wada K., Kita S., Tsuzaki K., Nose H., Kataoka M., Shimizu S. (2005). J. Biotechnol..

[cit10] Kobayashi M., Shinohara M., Sakoh C., Kataoka M., Shimizu S. (1998). Proc. Natl. Acad. Sci. U. S. A..

[cit11] Honda K., Tsuboi H., Minetoki T., Nose H., Sakamoto K., Kataoka M., Shimizu S. (2005). Appl. Microbiol. Biotechnol..

[cit12] Tang Y.-X., Sun Z.-H., Hua L., Lv C.-F., Guo X.-F., Wang J. (2002). Process Biochem..

[cit13] Kesseler M., Friedrich T., Höffken H. W., Hauer B. (2002). Adv. Synth. Catal..

[cit14] Zhang X., Xu J.-H., Xu Y., Pan J. (2007). Appl. Microbiol. Biotechnol..

[cit15] Chen B., Fan L.-Q., Xu J.-H., Zhao J., Zhang X., Ouyang L.-M. (2010). Appl. Microbiol. Biotechnol..

[cit16] Honda K., Kataoka M., Shimizu S. (2002). Biotechnol. Bioprocess..

[cit17] Thompson J. D., Gibson T. J., Higgins D. G. (2003). Curr. Protoc. Bioinf..

[cit18] Robert X., Gouet P. (2014). Nucleic Acids Res..

